# Construction of a breast cancer prognosis model based on alternative splicing and immune infiltration

**DOI:** 10.1007/s12672-022-00506-0

**Published:** 2022-08-21

**Authors:** Dongni Zhang, Wenping Lu, Zhili Zhuo, Heting Mei, Xiaoqing Wu, Yongjia Cui

**Affiliations:** Oncology Department, China Academy of Chinese Medical Sciences Guang’anmen Hospital, Beijing, China

**Keywords:** Breast cancer, Alternative splicing, Immune infiltration, Prognosis, Risk model, Tumor microenvironment

## Abstract

**Background:**

Breast cancer (BC) is the most common malignancy among women in the world. Alternative splicing (AS) is an important mechanism for regulating gene expression and producing proteome diversity, which is closely related to tumorigenesis. Understanding the role of AS in BC may be helpful to reveal new therapeutic targets for clinical interventions.

**Methods:**

RNA-seq, clinical and AS data of TCGA-BRCA were downloaded from TCGA and TCGA SpliceSeq databases. AS events associated with prognosis were filtered by univariate Cox regression. The AS risk model of BC was built by Lasso regression, random forest and multivariate Cox regression. The accuracy of the AS risk model and clinicopathological factors were evaluated by time-dependent receiver operating characteristic (ROC) curves. The significant factors were used to construct the nomogram model. Tumor microenvironment analysis, immune infiltration and immune checkpoint analysis were performed to show the differences between the high and low AS risk groups. The expression differences of genes of AS events constituting the risk model in tumor tissues and normal tissues were analyzed, the genes with significant differences were screened, and their relationship with prognosis, tumor microenvironment, immune infiltration and immune checkpoint were analyzed. Finally, Pearson correlation analysis was used to calculate the correlation coefficient between splicing factors (SF) and prognostic AS events in TCGA-BRCA. The results were imported into Cytoscape, and the associated network was constructed.

**Results:**

A total of 21,232 genes had 45,421 AS events occurring in TCGA-BRCA, while 1604 AS events were found to be significantly correlated with survival. The BRCA risk model consisted of 5 AS events, (TTC39C|44853|AT*− 2.67) + (HSPBP1|52052|AP*− 4.28) + (MAZ|35942|ES*2.34) + (ANK3|11845|AP*1.18) + (ZC3HAV1|81940|AT*1.59), which were confirmed to be valuable for predicting BRCA prognosis to a certain degree, including ROC curve, survival analysis, tumor microenvironment analysis, immune infiltration and immune checkpoint analysis. Based on this, we constructed a nomogram prediction model composed of clinicopathological features and the AS risk signature. Furthermore, we found that MAZ was a core gene indicating the connection of tumor prognosis and AS events. Ultimately, a network of SF-AS regulation was established to reveal the relationship between them.

**Conclusions:**

We constructed a nomogram model combined with clinicopathological features and AS risk score to predict the prognosis of BC. The detailed analysis of tumor microenvironment and immune infiltration in the AS risk model may further reveal the potential mechanisms of BC recurrence and development.

**Supplementary Information:**

The online version contains supplementary material available at 10.1007/s12672-022-00506-0.

## Introduction

Globally, breast cancer (BC) ranks first in incidence [1], and second in mortality just after lung cancer in women [2]. With early diagnosis and treatment, the mortality rate of BC has declined. However, the morbidity rate is still increasing year over year. Up to January 1, 2019, there were about 3.8 million BC patients in the United States [2], In China, there were about 278,900 newly diagnosed cases of BC and about 66,000 deaths in 2014 [3, 4]. At present, the main treatments for BC include surgery, radiotherapy, chemotherapy, endocrine therapy, targeted therapy, immunotherapy, etc. [5]. However, drug resistance to a plethora of therapeutic interventions makes it difficult to achieve ideal therapeutic effect. Apparently, deeper understanding of the mechanism of drug resistance, pathogenesis of the malignancy, and therapeutic targets is critical to further increase the efficacy of these various therapies.

Alternative splicing (AS) is a posttranscriptional process that leads to one single gene encoding multiple proteins. It has been proven that cancer cells have general, cancer type-specific and subtype-specific diversity during splicing, which may have prognostic value and facilitate features of cancer progression, including the cancer immune response, epithelial-to-mesenchymal transition, and resistance to chemotherapeutic drugs [6–8]. These splicing changes are usually associated with the occurrence of cancer-driven mutations in genes encoding core components or regulators of splicing mechanisms [9]. TCGA SpliceSeq (http://bioinformatics.mdanderson.org/TCGASpliceSeq) is a database used to analyze AS events in protein coding genes based on TCGA data. The percent-splice-in (PSI) value is a quantitative index that can describe the probability of occurrence of an AS event. By downloading the PSI data of all AS events of patients in TCGA-BRCA from TCGA SpliceSeq and combining with the RNA-seq data and survival information of TCGA-BRCA, AS events related to prognosis can be obtained, and patients can be divided into high-risk and low-risk groups according to these AS events. By analyzing the RNA-seq information of the two groups of patients, we can speculate the potential role and possible mechanism of these prognosis-related AS events in BC.

The process of tumor formation is not only the self-reproduction and accumulation of tumor cells but also the result of the combined effects of tumor cells and adjacent immune cells, stromal cells and extracellular matrix, just as the growth of seeds requires soil nourishment. All these tumor formation participants constitute the tumor microenvironment (TME), which is a complex and dynamic cellular community [10]. Stromal cells, including cancer-associated fibroblasts (CAFs), endothelial cells, adipose cells, etc. are important components of the formation and progression of solid tumors and affect the efficacy of antitumor therapy [10–12]. As the guardian of the human body, immune cells are responsible for clearing pathogens and mutated cells in the body (tumor cells and senescent cells) to maintain healthiness. However, immune cells in the TME are prone to dysfunction due to inadequate nutrition and remodeling of the hostile TME, which remains a pivotal barrier for cancer immunotherapies and is related to tumor progression and poor prognosis [13, 14]. The interplay between the TME and tumor cells is a critical contributor to immune evasiveness, physiological hardiness and tumor recurrence and metastasis. In recent years, the most breakthrough tumor therapeutic drugs have been immune checkpoint inhibitors (ICIs), represented by PD-1/PD-L1 monoclonal antibodies. The major difference between ICI and traditional radiotherapy and chemotherapy is that its main target is not tumor cells but immune cells in the TME. Nevertheless, only a few patients can benefit from it, and increasing evidence indicates that the efficacy of ICI is reliant on a strong antitumor immune response, which is likely to be damaged in most tumors [15, 16]. The prospective therapeutic concepts in BC have gradually transformed into individualized therapeutic schedules on the basis of tumor biology and early response. It is therefore particularly urgent to discover new potential biomarkers to screen the benefits of existing treatment methods and explore new therapeutic targets and ways to enhance curative effects.

Due to the rapid development of bioinformatics, we are now able to estimate proportions of various types of cells in TME based on RNA-seq data using R packages. The “estimate” package can infer the fraction of stromal and immune cells in tumor specimens by gene expression signatures based on single sample gene set enrichment analysis (ssGSEA), stromal score (capturing the presence of stromal cells in tumor tissue), immune score (infiltration of immune cells in tumor tissue) and ESTIMATE score (immune and stromal integrated score) are calculated [17]. Higher tumor purity and lower immune cell abundance may be associated with poor prognosis and immunotherapeutic response. Moreover, by using “CIBERSORT” and the gene signatures of 22 immune cells obtained from its official website, the proportion of different immune cells in tumor samples can be assessed [18]. Different immune cells play different roles in tumorigenesis; therefore, looking for different immune cells may provide references for the study of tumorigenesis, the mechanism of AS events in tumors, and the immunotherapeutic response.

In this study, we filtered AS events significantly related to BC prognosis to establish an AS-associated risk model using the transcriptome and AS data downloaded from TCGA-BRCA and TCGA SpliceSeq databases. According to the model, the BC patients can therefore be classified into AS-high and AS-low risk groups. Subsequently, we performed univariate and multivariate Cox regression analyses of clinical information of TCGA-BRCA to screen for clinical features correlated with prognosis and generate a nomogram that can predict survival more accurately. To further precisely model the AS events and predict their effect on BC progression, we performed TME, immune infiltration and immune checkpoints analyses to explore how and why these AS events are associated with BC prognosis.

## Materials and methods

### TCGA data acquisition and preparation

The transcriptome data and clinical information of TCGA-BRCA were acquired from TCGA (The Cancer Genome Atlas, https://portal.gdc.cancer.gov/) database, which contains the mRNA-seq data of 56,461 genes’ expression in 1109 BC samples and 113 normal breast tissue samples from 1092 cases. The PSI (percent-splice-in) values of AS (alternative splicing) were obtained and calculated from the TCGA SpliceSeq database (https://bioinformatics.mdanderson.org/TCGASpliceSeq/PSIdownload.jsp), which consists of 7 types of AS events as follows: AA (alternate acceptor site), AD (alternate donor site), AP (alternate promoter), AT (alternate terminator), ES (exon skip), ME (mutually exclusive exons), and RI (retained introns) [9]. A total of 1195 samples and 45,421 human SF (splicing factor) genes were downloaded by the screening criterion of PSI value > 75%. The IDs were in the form “MAZ|35942|ES”, among which “MAZ” is the gene symbol, “35942” is the ID number, and “ES” represents the splicing type. The intersection and distribution among all types of AS were displayed by an upset plot.

### Obtainment of survival-related AS events

All data were processed using R (version: 4.1.2), and the UpSetR [19] (version: 1.4.0) package was used for the visualization of multiple sets. Univariate Cox regression analysis was performed by using the survival (Version: 3.2-13) R package, with p < 0.05 as the criterion, to obtain survival-related AS events. The top 20 most significant AS events of the 7 types were displayed by bubble plots through the ggplot2 (Version: 3.3.5) package, while the strength of the association between AS events and prognosis of BC was drawn into a volcano plot.

### Construction of the breast cancer AS event risk model

The survival-related AS events were analyzed by LASSO (least absolute shrinkage and selection operator) regression and random forest using the glmnet [20] (Version: 4.1-3) and survival (Version: 3.2-13) packages. The intersection set of AS events filtered by the two algorithms was calculated by multivariate Cox analysis to construct the risk model as follows: Riskscore = ${\sum }_{i}^{n}PSI\beta i$, where β stands for the regression coefficient. The samples could be split into high-risk and low-risk groups taking the median value of the risk score as the dividing line. Subsequently, the survival difference between the two groups was displayed by a Kaplan–Meier (KM) curve, while the relationship between the risk score and BC prognosis was evaluated by a PSI value heatmap, risk score curve and survival status diagram.

### Establishment of a nomogram in accordance with the AS event risk model

The clinicopathological features and risk score of the TCGA-BRCA cohort were analyzed by univariate Cox regression and multivariate Cox regression using the survival (Version: 3.2-13) R package, with p < 0.05 as the criterion. The HR and regression coefficient for each prognostic feature were calculated. The accuracy of the risk model and clinicopathological features were evaluated by ROC curves. The rms (Version: 6.2-0) R package was used to construct a nomogram predicting the 3-year, 5-year, and 10-year survival of BC patients, which consolidated the clinicopathological features and AS event signatures.

### Analysis of the tumor microenvironment and immune infiltration in accordance with the risk score

The ESTIMATE score, immune score, stromal score, and tumor purity of high- and low-risk patients in TCGA-BRCA were calculated by using the estimate (https://bioinformatics.mdanderson.org/estimate/rpackage.html) R package and visualized in violin plots. The R package “e1071” (Version: 1.7-9) was loaded as a precondition for CIBERSORT [18], which was used to estimate the different abundances of 22 immune cells in the high- and low-risk groups. The outcomes were shown in box plots. After that, the expression levels of immune genes in TCGA-BRCA were analyzed by the ssGSEA algorithm using the GSVA [21] (Version: 1.42.0), limma [22] (Version: 3.50.0), and GSEABase (Version: 1.56.0) packages, and the differences in immune scores between the high- and low-risk groups were shown in box plots. Limma, corrplot (Version: 0.92), ggpubr (Version: 0.4.0) and ggExtra (Version: 0.9) packages were used to analyze the correlation of immune checkpoint genes and draw the correlation scatter plot. The immune checkpoint genes with significant differences in expression between the high- and low-risk groups were displayed in a box plot.

### Screening and analysis of genes related to prognosis based on the AS risk score

The expression data of 113 normal breast tissue samples and 1109 tumor samples in TCGA-BRCA were analyzed by the limma package, and the differentially expressed genes significantly associated with survival were screened with logFC > 1 and p < 0.05 as the filter condition. Subsequently, the relationship between the expression levels of these genes and survival, tumor purity, immune infiltration and immune checkpoints was analyzed.

### Construction of the correlation network between SF genes and AS events

A total of 404 SF genes were obtained from the literature [23]. The gene expression data of TCGA-BRCA, the SF gene list, and the significant variable AS events obtained from the previous univariate Cox regression analysis were used as the input files for limma package analysis. Pearson correlation was used for analysis, and the filtering standard was set as absolute values of correlation coefficient > 0.6 and p value < 0.001. The analysis results were imported into Cytoscape (version: 3.7.2) to demonstrate the correlation network of SF genes and AS events in BC.

## Results

### Preliminary analysis of AS events in TCGA-BRCA

**Fig. 1:**
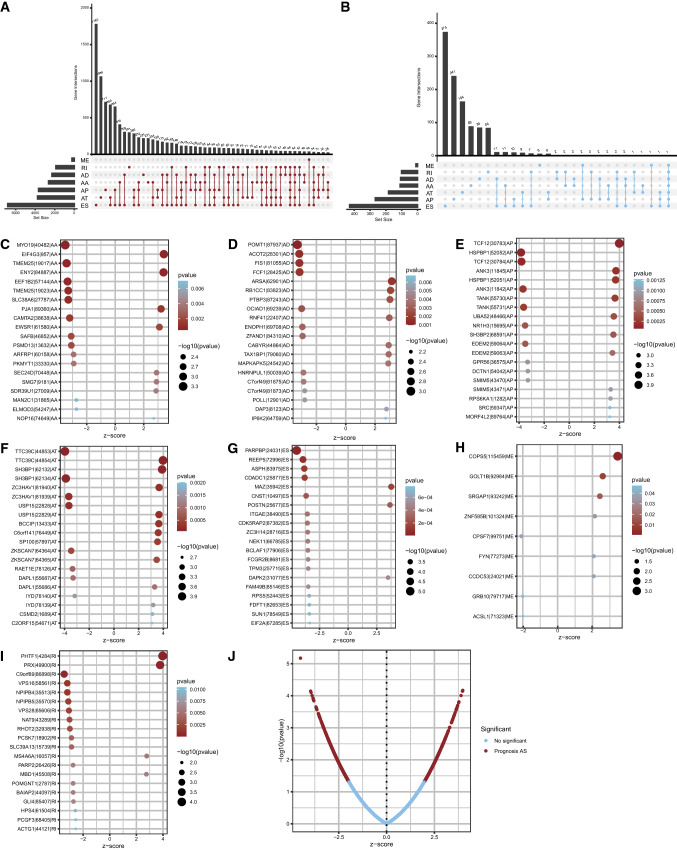
**A** Total analysis of AS events in TCGA-BRCA. **A** The intersections of 7 types of AS events in BC displayed by an upset plot; **B** UpSet plot of the intersections of 7 types of AS events significantly associated with survival in BC filtered by univariate Cox regression analysis (p < 0.05); **C**–**I** bubble plots of the AS events significantly associated with survival in BC; **J** the strength of association between AS events and BC shown by volcanic plot

A total of 21,232 genes had 45,421 AS events occurring in 1195 samples in TCGA-BRCA (Fig. 1A), and the incidence of AS events in BC was relatively high. Among these events, ES was the most predominant AS event, with 17,702 occurring in 6812 genes, and ME was the least predominant event, with 233 events in 227 genes. The survival data of patients in TCGA-BRCA were combined with their AS event occurrence data and subjected to univariate Cox regression to preliminarily acquire survival-related AS events. The results were shown in the upset plot in Fig. 1B. A total of 1064 AS events in 1081 TCGA-BRCA patients were screened out. The top 20 significant prognostic AS events of each AS types were shown in bubble plots (if there were fewer than 20 events, all significant events would be displayed) (Fig. 1C–I). The strength of the association between AS events and BC was shown by a volcano plot (Fig. 1J).

### Screening for AS events associated with breast cancer prognosis

**Fig. 2:**
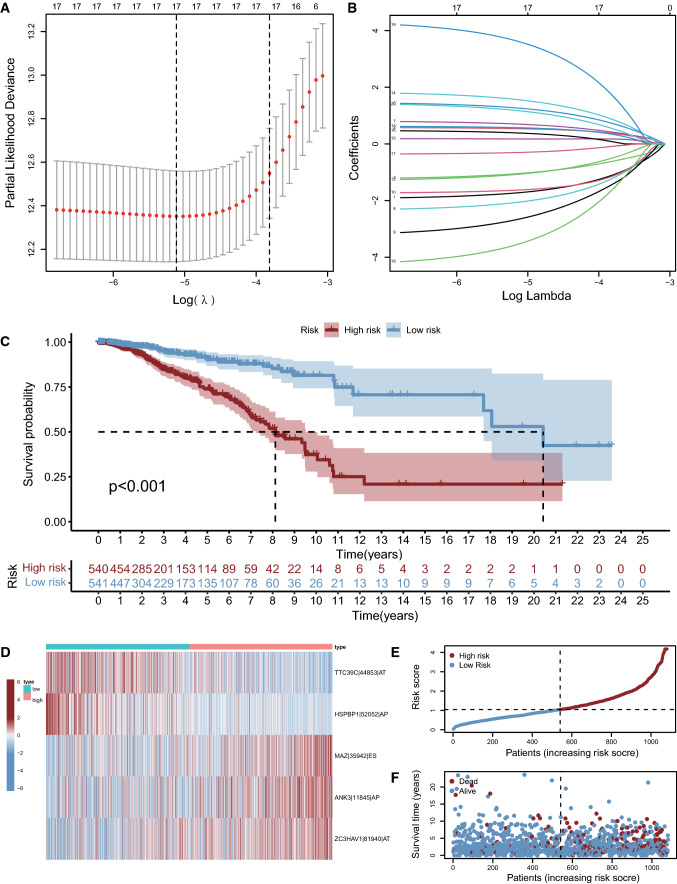
**A** As lambda grows, the regularization term has a greater effect, more coefficients will be zero, and fewer variables in the model remain. **B** Lasso regression analysis obtained the optimal number of AS events to construct the risk model. **C** KM survival curve verified that there was a significant difference in survival between high-risk and low-risk patients. **D** Heatmap of the PSI value of 5 AS events in the high- and low-risk groups. **E** The risk scores of AS signatures in patients of TCGA-BRCA. **F** The distribution of survival time of AS signatures in TCGA-BRCA patients

The data of 1064 AS events in 1081 TCGA-BRCA patients were used for Lasso regression and random forest analysis to narrow the candidate AS events down. Lasso regression obtained 17 AS events (Fig. 2A, B), while random forest obtained 5. Finally, 5 AS events were identified as the intersection of these two algorithms and the ultimate components of the AS prognostic model of BC. The correlation coefficients were calculated by multivariate Cox regression (Table 1). The integrated prognostic signature consisted of (TTC39C|44853|AT*− 2.67) + (HSPBP1|52052|AP*− 4.28) + (MAZ|35942|ES*2.34) + (ANK3|11845|AP*1.18) + (ZC3HAV1|81940|AT*1.59). A higher risk score was considered to be linked with worse prognosis. The incidents of AS events with positive correlation coefficients were believed to be correlated with disappointing outcomes; in contrast, the occurrence of AS events with negative correlation coefficients were regarded as protective factors of BC and might correlate with a pleasant ending. The risk score of TCGA-BRCA patients could be obtained by calculating the occurrence of AS events according to the above formula. The median risk score was taken as the dividing point, and TCGA-BRCA patients were then subdivided into high-risk and low-risk groups. The K-M survival curve verified that the low-risk group had a significantly better prognosis than the high-risk group, p < 0.001 (Fig. 2C). The PSI values of 5 AS events in the high-risk and low-risk group were shown in a heatmap (Fig. 2D), the PSI values of TTC39C|44853|AT and HSPBP1|52052|AP were higher in the low-risk group, while the PSI values of MAZ|35942|ES, ANK3|11845|AP, and ZC3HAV1|81940|AT were higher in the high-risk group. The relationship of the risk score and prognosis in the high-risk and low-risk groups was displayed by risk score curves (Fig. 2E) and survival scatter plots (Fig. 2F), which could be seen that with the higher risk score, the number of dead patients increased. These results indicated that the BC risk model composed of 5 AS events can predict the survival of BC to a certain extent.

**Table 1:** The regression coefficient of 5 AS events in the breast cancer risk model

ID	Coefficient	HR	HR.95 L	HR.95 H	p value
TTC39C|44853|AT	− 2.66685150553005	0.0694706091675454	0.0152247875683456	0.316993949271523	0.000574554918772557
HSPBP1|52052|AP	− 4.2838175189376	0.013789918201263	0.00159124112899631	0.119505360018862	0.000101017804163208
MAZ|35942|ES	2.33644819154652	10.3444298029252	1.54792689941532	69.1293807143387	0.0159184146461527
ANK3|11845|AP	1.18206915323645	3.26111497324261	1.74615196850633	6.09046123162134	0.000208120437009903
ZC3HAV1|81940|AT	1.585753619274	4.88296989246445	1.43303039862465	16.6384432553545	0.0112393488811907

### Construction of a nomogram model in accordance with the AS signature

**Fig. 3:**
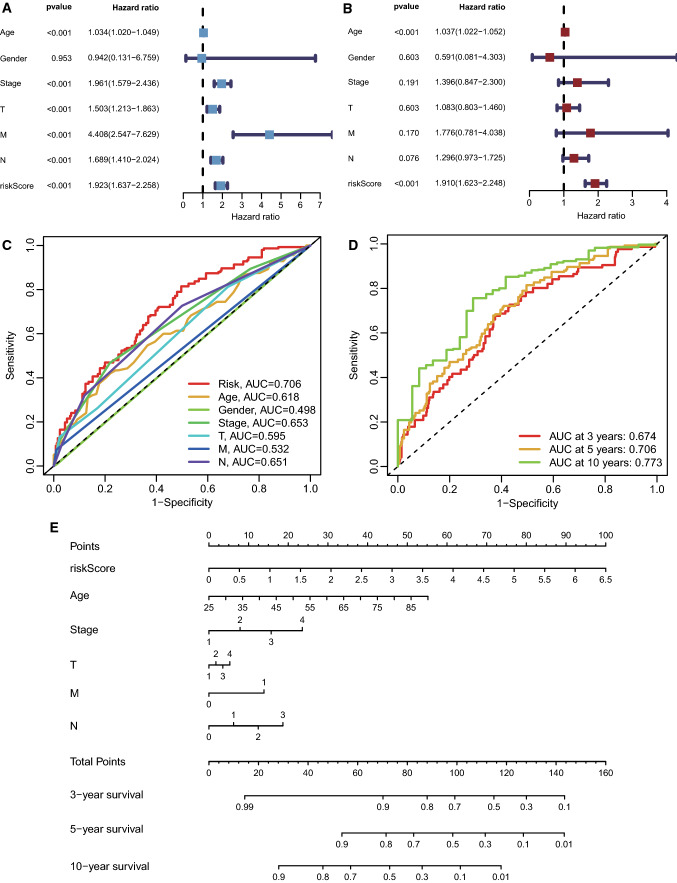
Construction of a nomogram model in accordance with the AS signature. **A** Univariate Cox regression analyses of the AS risk model and clinicopathological data; **B** multivariate Cox regression analysis of the AS risk model and clinicopathological data; **C** The prognostic value of the AS risk model and clinicopathological factors evaluated using ROC curves at 5 years; **D** the prognostic value of the AS risk model was evaluated using ROC curves at 3, 5, and 10 years; **E** the nomogram model was constructed to predict the 3-, 5-, and 10-year survival of breast cancer patients

The clinical data of 921 patients with complete clinical information in TCGA-BRCA were successively computed by univariate and multivariate Cox regression to filter the clinical factors related to prognosis. In univariate Cox regression analysis, age, tumor stage, T, N, M grade and AS risk score were significantly related to prognosis, p < 0.001 (Fig. 3A). Due to the low proportion of male patients (11/921), we were conjectured to be unable to perform reliable analysis. In multivariate Cox regression, age and risk score still had an intense relationship with prognosis, p < 0.001 (Fig. 3B), suggesting they could be independent prognostic predictors for BC. To further inspect the utility of these clinical factors, ROC curves at 5 years were drawn, and the area under the curve (AUC) values were calculated. Except for sex, the AUC of the other factors was > 0.5 (Fig. 3C), implying a potential value in the prognosis of BC. We could catch sight from the ROC curves at 3, 5, and 10 years of the AS risk model, the exactitude of which gradually increased over time, indicating that this AS risk model might have some significance for the long-term prognosis of BC (Fig. 3D). Next, we excluded sex as no significant relationship with prognosis and used the rms package to draw a nomogram according to the remaining clinicopathological features and AS risk score to predict the survival rate at 3, 5, and 10 years in breast cancer (Fig. 3E).

### Analysis of the tumor microenvironment and immune infiltration

**Fig. 4:**
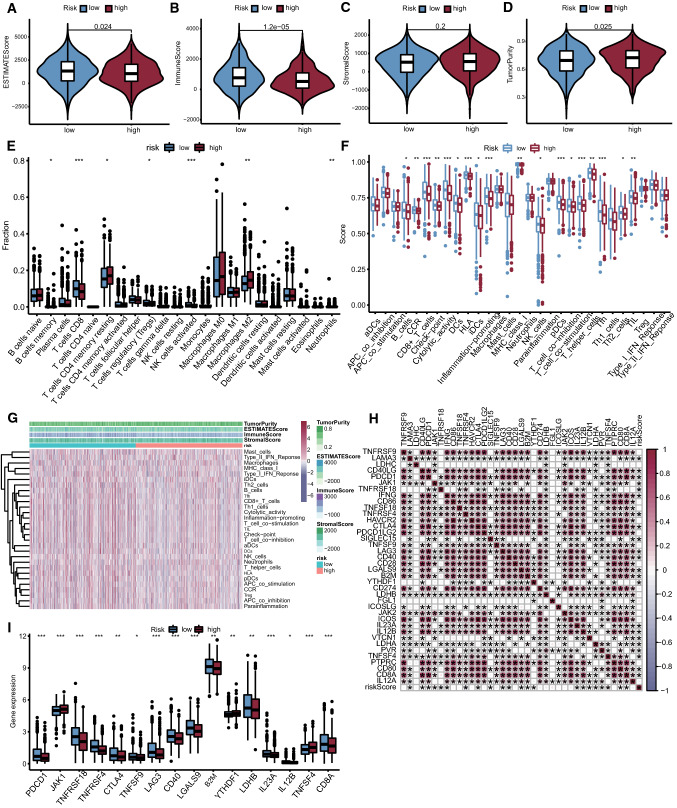
Analysis of the tumor microenvironment and immune infiltration. **A**–**D** Violin plots of ESTIMATE score, immune score, stromal score, tumor purity differences between high- and low-risk groups; **E** Box plot of CIBERSORT results; **F** Box plot of ssGSEA results; **G** Heatmap of comprehensive results of ESTIMATE results and ssGSEA results; **H** Correlation scatter plot of 38 immune checkpoint differences between high- and low-risk groups; **I** Box plot of significantly different immune checkpoints between high- and low-risk groups. (*p < 0.05, **p < 0.01, ***p < 0.001)

Taking the median AS risk score as the cutoff value, the patients in TCGA-BRCA were divided into a high-risk group and a low-risk group. The “estimate” and “limma” packages were used to determine the scores of stromal cells and immune cells in the expression profile data of TCGA-BRCA, therefore estimating the tumor purity and immune cell infiltration of each tumor sample. As shown in the figures (Fig. 4A, D), ESTIMATE score, and tumor purity of the low-risk group were lower than those of the high-risk group (p < 0.05), indicating that the tumor samples in the high-risk group had higher tumor purity. This result proved that the high-risk score was associated with poor outcomes, and the occurrence rate of model AS events affected the progression of BC to some extent. Meanwhile, the low-risk group had a significantly higher immune score (Fig. 4B). On the other hand, there was no significant difference in stromal score between the two groups (Fig. 4C), suggesting that these 5 AS events might affect BC by influencing immune infiltration rather than through stromal cells. To further reveal how these 5 AS events affect immune infiltration, two mainstream computing immune infiltration methods, CIBERSORT and ssGSEA, were used for analysis. The operating result of CIBERSORT (Fig. 4B, D) discovered higher fractions of memory B cells, CD8 T cells, regulatory T cells (Tregs), and natural killer (NK) cells activated in the low-risk group, while the high-risk group had higher proportions of resting memory CD4 T cells, M2 macrophages and neutrophils. By using the ssGSEA method, it was found that there were significant differences in 18 of 29 immune cells (Fig. 4B, F). The intersection of these two processes was higher ratios of CD8 + T cells and NK cells in the low-risk group, implying the possibility that the rise of these 5 AS events might regulate the accumulation of these 2 immune cells, thus affecting the TME and the therapeutic effect. The consolidated ssGSEA and “estimate” results were displayed in a heatmap (Fig. 4G). The Spearman method was used to analyze the correlation between the expression level of 38 immune checkpoints and the AS risk score, and the correlation coefficient was calculated. Sixteen immune checkpoints were found to be significantly related to the AS risk score (Fig. 4H). The low-risk group had higher expression levels of PDCD1, TNFRSF18, TNFRSF4, CTLA4, TNFRSF9, LAG3, CD40, LGALS9, B2M, LDHB, IL23A, IL12B, and CD8A and lower expression levels of JAK1, YTHDF1, and TNFSF4 (Fig. 4I). This suggested that the occurrence of 5 AS events might affect the expression of immune checkpoints and that the AS risk score might be a promising prognostic indicator of ICI treatment, but its specific mechanism and sensitivity still need to be further studied.

### MAZ was related to prognosis based on the AS risk model

**Fig. 5:**
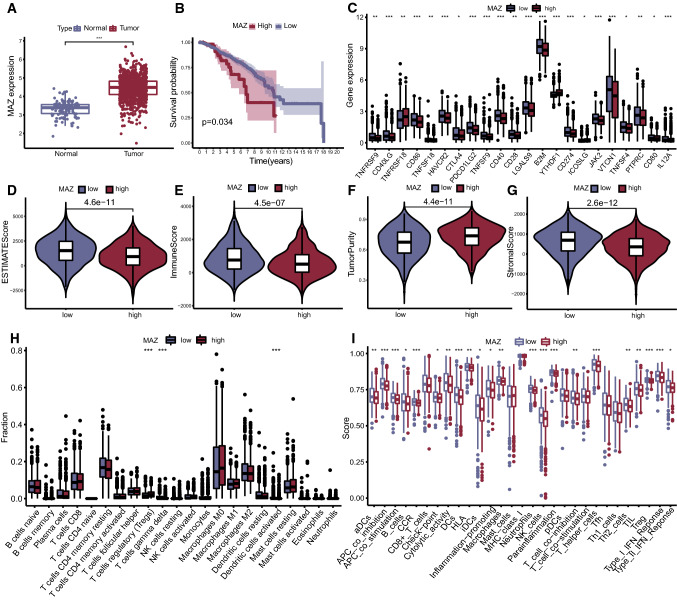
Screening and analysis of the prognosis-related gene MAZ. **A** Expression difference of MAZ between tumor and normal tissue in TCGA-BRCA; **B** KM survival curve of high and low MAZ expression in TCGA-BRCA; **C** immune checkpoint difference between high and low MAZ expression patients in TCGA-BRCA; **D**–**G** Violin plots of ESTIMATE score, immune score, tumor purity, stromal score differences between high and low MAZ expression patients; H) Box plot of CIBERSORT results in high and low MAZ expression; **I** Box plot of ssGSEA results in high and low MAZ expression. (*p < 0.05, **p < 0.01, ***p < 0.001)

The 5 genes (TTC39C, HSPBP1, MAZ, ANK3 and ZC3HAV1) where model AS events occurred were analyzed for the differential expression level between normal breast tissue and tumor tissue. A statistically significant difference was found in the expression level of MAZ between normal and BC tissues (Fig. 5A). The K–M survival curve also confirmed a significant correlation between its expression level and BC prognosis, and the survival of BC patients with lower expression was better than that of patients with high expression (Fig. 5B). Immune checkpoint analysis demonstrated that the expression level of a single gene, MAZ, could be connected to the expression differences in 22 immune checkpoint genes (Fig. 5C). TME analysis by using “estimate” stated that the higher expression level of MAZ was related to higher tumor purity and a lower percentage of immune cells and stromal cells (Fig. 5D–G), which provided additional proof of its prognostic value. The results of immune infiltration analysis of CIBERSORT and ssGSEA also showed that there were certain differences in the types and proportion of immune cells between the high and low expression of MAZ groups (Fig. 5H, I). The above results suggested that AS events of MAZ might be the core of the 5 AS events that consisted of the BC risk model, leading to TME changes and immune infiltration in BC patients.

### Construction of a network of prognostic SF-AS in TCGA-BRCA

**Fig. 6:**
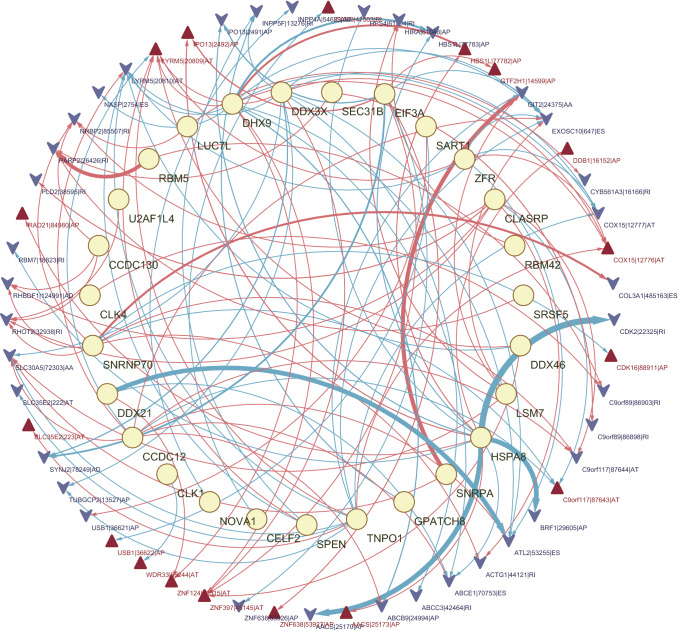
Network of prognostic SF-AS in TCGA-BRCA. The yellow ellipse represents splicing factors, the red regular triangle represents risk AS events, and the blue inverted arrow represents protective AS events. The red/blue line represents positive/negative regulation between AS events and SF, and the thickness of the line represents the correlation strength

Furthermore, to explore the upstream regulatory network of these AS events related to BC prognosis, the correlation between splicing factor (SF) genes and prognosis-related AS events screened by the univariate Cox regression above was calculated by the Pearson method. Filtered by the standard with absolute values of correlation coefficient > 0.6 and p value < 0.001, 27 SF was associated with 120 prognostic AS events (supplementary table 1). The network was constructed by Cytoscape (Fig. 6), and the relationship between SF genes and AS events was not only one-to-one but many-to-one or many-to-many. In addition, SFs have positive and negative adjustments for high- and low-risk events. This network diagram offered some references for exploring the upstream mechanism of AS events related to the prognosis of BC, but its specific regulatory mechanism still needs further experimental research.

## Discussion

The complex and important role of AS in tumorigenesis and progression has been widely studied and confirmed. As one of the important processes in the regulation of protein diversification, the pattern of splicing is often altered in cancer. A number of cancer molecular subtypes heavily rely on the splicing function of cell survival, since the mutations of genes encoding spliceosome proteins and those affecting key genes associated with cancer are enriched in cancer [24, 25]. Our study also found significant differences in the incidence of AS events between patients with better survival and those with poor survival in TCGA-BRCA. These results indicate that the change in certain AS events may be related to a series of changes in the tumor process and can be used as a potential biomarker. Targeting specific key AS events in cancer may be a promising direction for treatment and prognosis. Studies are currently trying to broaden the potential target space for immunotherapy of some cancers untreatable by focusing on alternative splicing events [25, 26]. Therefore, our research aimed to determine the valuable AS events associated with breast cancer prognosis and to explore their potential mechanism through analysis of the tumor microenvironment and immune infiltration to provide some references for follow-up research.

A breast cancer risk model consisting of 5 AS events was constructed in our study, which was risk score = (TTC39C|44853|AT*− 2.67) + (HSPBP1|52052|AP*− 4.28) + (MAZ|35942|ES*2.34) + (ANK3|11845|AP*1.18) + (ZC3HAV1|81940|AT*1.59). There are few reports about TTC39C (tetratricopeptide repeat domain containing 39c), which may be related to muscle cell differentiation through the MAP kinase and hedgehog signaling pathways [27] and may be a new sensitive and specific gene expression marker in lung cancer harboring STK11 mutations [28]. Therefore, the specific mechanism of TTC39C in breast cancer remains to be further studied.

HSPBP1 (heat shock protein 70-binding protein 1) is a member of the eukaryotic protein family identified as a nucleotide exchange factor for Hsp70 and therefore influences protein quality control [29, 30], which has already been studied in HIV-1, neuropathology, atherosclerosis and other fields [31–33]. Evidence has shown that the high expression of HSPBP1 may be related to cytotoxic action and tumor aggressiveness, which can be a protective factor of tumors [34–36]; this is also consistent with our results.

ANK3 (ankyrin 3) encodes a variety of ankyrin-G isoforms, which play an important role in neural development and are well-explored in psychiatric disorders [37, 38]. There are few reports on ANK3 in cancer, and one showed that ANK3 can be used as a prognostic factor for two primary histologic subtypes of cutaneous melanoma [39] and may be associated with breast cancer prognosis by regulating the androgen receptor signaling pathway [40]. Our research also shows that it is associated with the prognosis of breast cancer, suggesting that its potential role in cancer needs to be studied.

ZC3HAV1 (zinc finger CCCH-type antiviral protein 1), also known as PPAR13 [poly (ADP-ribose) polymerase-13] and ZAP (zinc-finger antiviral protein), is a PARP family member of RNA-binding proteins and has important functions in the cellular response to stress [41]. Studies have shown that it increases cell sensitivity to apoptosis [42], participates in facilitating DNA repair and promoting tumorigenesis of breast cancer [43], and facilitates metastasis and proliferation in pancreatic cancer [44], suggesting that it can be involved in malignant transformation and the development of cancer.

MAZ (Myc-associated zinc-finger protein) is an oncogene supported by many studies. It is considered to activate KRAS transcription and may induce the expression of the proto-oncogene MYB, participating in facilitating aerobic glycolysis and other functions and is therefore involved in the tumor progression and metastasis of several cancer types [45–48]. There is also evidence that in breast cancer, MAZ may have a dual function in regulating the development and progression of basal-like breast cancer in different stages and promote the malignant phenotypes of triple-negative breast cancer cells [49, 50]. Our research confirms the results of previous studies. The expression of MAZ in breast cancer tissues was significantly higher than that in adjacent normal tissues, and the survival rate of patients with high MAZ expression was significantly lower than that of patients with low expression. In addition, the high expression of MAZ is also related to high tumor purity and immune infiltration.

We also found that the risk scores of 5 AS model events were closely related to the proportion of immune cells. Using two mainstream immune infiltration assessment methods, CIBERSORT and ssGSEA, it was found that the intersection differences in immune cells were CD8 + T cells and NK cells. The proportion of both cell types in the low-risk group was higher than that in the high-risk group. In tumor progression, CD8+ T cells are the key mediators of cytotoxic effector function, play an important part in the adaptive immunity of the body and are one of the main effector cells of tumor adoptive immunotherapy [51, 52]. However, in the setting of cancer, chronic antigen exposure in TME cues contributes to T cell exhaustion and dysfunction, which is a major barrier to anticancer immunotherapies [52, 53]. NK cells have an important position in the process of antitumor innate immunity and are highly heterogeneous in the TME; they can inhibit tumor cell proliferation and migration to distant colonization by exerting natural cytotoxicity [54, 55]. They have also been discovered to have a role in modulating adaptive immune responses as well as other related pathways by producing cytokines [56]. Nevertheless, the interaction within the TME can lead to immunosuppression and tumor escape by exposing NK cells to inhibitory molecules produced by tumor cells [57]. The difference in the proportion of these two immune cells not only confirms the prediction fidelity of the AS model but also provides some ideas for exploring the mechanism of how these AS events are related to the prognosis of BC. However, the regulatory relationship between these AS events and CD8 + T cells and NK cells remains to be further studied.

As the upstream of AS events, the SF gene indirectly affects the occurrence and development of cancer by affecting the AS process [58]. Our previous research found some AS events associated with immune cells and immune checkpoints, so the SF gene related to these AS events may be a potential therapeutic target. By targeting the SF gene, we may be able to adjust the occurrence of corresponding AS events, which may have an impact on the TME and immunity and improve the intensity of autoimmunity and the effect of immunotherapy.

Our study preliminarily corroborated the association between AS events and breast cancer prognosis and found that the occurrence of 5 AS events constituting the risk model was related to the TME, the expression of immune checkpoint genes and immune cell infiltration, which may lead to differential responses to immunotherapy. However, there are still many deficiencies, and there is a lack of experiments to verify the regulatory effect of the screened SF gene on the 5 AS events and the specific mechanism of the 5 AS events on the modulation of immune infiltration. More independent clinical trials are needed to recruit enough patients to examine the prognostic value of this risk model.

## Conclusions

We constructed a risk model associated with the prognosis of breast cancer consisting of 5 AS events. The AUC values of this model predicting TCGA-BRCA patients at 3, 5, and 10 years were 0.674, 0.706, and 0.773, respectively, suggesting a certain prognostic value in BC. These AS events were also found to be associated with the TME, immune infiltration and immune checkpoints, suggesting that their occurrence ratio may be related to the response to immunotherapy and ICI treatment, thus affecting the progression of BC. These AS events may provide a reference for screening new therapeutic targets, prognoses and therapeutic biomarkers for breast cancer.

## Supplementary Information

Supplementary material 1 (XLSX 16.7 kb) 

## Data Availability

All data in our study were available from the TCGA database (http://cancergenome.nih.gov/) and TCGA SpliceSeq database (https://bioinformatics.mdanderson.org/TCGASpliceSeq/PSIdownload.jsp).

## Abbreviations

BC: Breast cancer
AS: Alternative splicing
SF: Splicing factor
PSI: Percent splice-in
ROC: Receiver operating characteristic
AUC: Area under the curve
TME: Tumor microenvironment
CAF: Cancer-associated fibroblasts
ICI: Immune checkpoint inhibitors
PD-1: Programmed cell death protein 1
ssGSEA: Single sample gene set enrichment analysis
TCGA: The Cancer Genome Atlas
LASSO: Least absolute shrinkage and selection operator
NK: Nature killer
TTC39C: Tetratricopeptide repeat domain containing 39c
HSPBP1: Heat shock protein 70-binding protein 1
ANK3: Ankyrin 3
ZC3HAV1: Zinc finger CCCH-type antiviral protein 1
MAZ: Myc-associated zinc-finger protein
